# Setting an Inlay

**Published:** 1904-05

**Authors:** T. V. Smith

**Affiliations:** Philadelphia.


### SETTING AN INLAY.

T. V. SMITH, PHILADELPHIA.

After the inlay has been inserted, scrape away excess cement and varnish with sandarach. Then apply paraffin, which will adhere for a much longer time to the sandarach than to the surface of inlay or tooth. Never have the rubber dam over the tooth when trying to match a shade, because when dry teeth are several shades lighter.

Excellent results may be obtained by the use of porcelain enamel paints, both by fusing on the surface of an inlay and under another modifying shade of enamel body.—*Extract International.*

